# Obstetric Nurses’ Approach to Evidence-Based Practice in Breastfeeding Within the Context of HIV: A Scoping Review

**DOI:** 10.3390/healthcare14091172

**Published:** 2026-04-27

**Authors:** Catarina Fonseca, Sara Palma, Mónica Antunes

**Affiliations:** 1 São João de Deus School of Nursing, Évora University, 7000-811 Évora, Portugal; catarinagoncalvesfonseca@gmail.com; 2 Unidade Local de Saúde São José, 1150-199 Lisbon, Portugal; 3 Nursing Research Innovation and Development Centre of Lisbon (CIDNUR), School of Nursing, University of Lisbon, 1600-096 Lisbon, Portugal; saraelisabetepalma@gmail.com; 4 Center for Interdisciplinary Health Research (CIIS), Portuguese Red Cross Health School, 1300-125 Lisbon, Portugal

**Keywords:** breastfeeding, evidence-based practice, HIV, mother-to-child transmission, obstetric nursing, women living with HIV

## Abstract

**Highlights:**

**What are the main findings?**

- The available evidence points to complex clinical, professional, and contextual challenges in translating HIV-breastfeeding guidelines into practice; interpretive analysis suggests these may involve professional uncertainty, the rapid evolution of guidance, and the negotiation of competing clinical priorities.
- Most research focuses on generic healthcare providers, obscuring the distinct contributions and educational needs of obstetric nurses.

**What are the implications of the main findings?**

- Training must integrate ethical formation, reflective practice, and advocacy skills, not just technical updates.
- Healthcare systems must invest in profession-specific research, institutional support, and professional leadership to empower obstetric nurses.

**Abstract:**

Background/Objectives: Human immunodeficiency virus remains a significant public health challenge, with breastfeeding contributing to the risk of mother-to-child transmission. Although antiretroviral therapy significantly reduces this risk, obstetric nurses face complex challenges in translating evolving guidelines into clinical practice. This scoping review aims to map existing scientific evidence on obstetric nurses’ approaches to evidence-based practice regarding breastfeeding in the context of HIV. Methods: Following the Joanna Briggs Institute methodology and PRISMA-ScR guidelines, a search was conducted across PubMed, Scopus and EBSCOhost (MEDLINE Complete, CINAHL Complete, Cochrane Central Register of Controlled Trials, and Nursing & Allied Health Collection: Comprehensive) for studies published in English and Portuguese between 2015 and 2025. Studies were included if they focused on the role of obstetric nurses, nurse-midwives, or midwives in infant-feeding practices for women living with HIV. Results: Eight studies were included, predominantly from sub-Saharan Africa, with additional evidence from Europe and Canada. Findings reveal that infant-feeding counseling is shaped by a complex interplay of clinical protocols and personal beliefs. Significant gaps in knowledge translation were identified. While nurses demonstrate high technical confidence in lactation support, their distinct professional contribution is often obscured by research that aggregates all healthcare providers. Conclusions: The challenge of supporting breastfeeding in the context of HIV extends beyond technical protocol adherence. It points to persistent gaps in knowledge translation, variability in counselling practices, and the influence of contextual and professional factors on guideline implementation. Strengthening care requires sustained investment in profession-specific education, institutional support, and evidence-informed practice frameworks that enable obstetric nurses to exercise informed clinical judgement.

## 1. Introduction

Human immunodeficiency virus (HIV) remains a critical global public health challenge, with transmission persisting across all nations [1]. Within the scope of women’s health, fostering an enabling environment is essential to uphold the sexual and reproductive health and rights of women living with HIV (WLWH) [2]. Such an environment encompasses access to strategies aimed at achieving safe and healthy pregnancies, given that HIV transmission from mother to child can occur during pregnancy, labour or delivery, but also afterwards through breastfeeding [2,3].

According to data released by the World Health Organization (WHO), approximately 1.3 million WLWH become pregnant each year worldwide [3]. In the absence of intervention, mother-to-child transmission (MTCT) rates range from 15% to 30% during the peripartum period, with breastfeeding increasing the cumulative risk by 5% to 15% [3]. Addressing this global health concern, it is imperative to implement comprehensive MTCT prevention programmes. These should prioritise access to HIV testing and the initiation of lifelong triple antiretroviral therapy (ART) for pregnant WLWH, coupled with adherence support [3]. Current findings demonstrate that when a mother living with HIV adheres to ART and achieves sustained viral suppression throughout pregnancy, delivery and breastfeeding, the risk of MTCT becomes less than 1% [4]. By the end of 2024, 1.1 million pregnant women with HIV had been identified [5]. Data from that year indicate that the annual number of children acquiring HIV through vertical transmission had dropped to 120,000, the lowest number since the onset of the epidemic [6].

To maximize HIV-free survival of HIV-exposed infants, it is essential to optimize infant-feeding practices [3,7]. The WHO states that national health authorities are responsible for defining the infant-feeding strategy that ensures the greatest chance of HIV-free survival [7]. This involves choosing between promoting breastfeeding alongside ART or recommending the total avoidance of breastfeeding, depending on local epidemiological, socioeconomic and cultural contexts, as well as the availability and quality of health services [7]. Breastfeeding is the gold standard for infant feeding due to its comprehensive nutritional, immunological, emotional and developmental benefits [8,9]. It also has several advantages for the mother, promoting postpartum recovery and reducing the risk of various diseases [8]. Mothers living with HIV and their infants may also benefit from the nutritional and immunological advantages of breastfeeding when effective ART and appropriate clinical monitoring are ensured [10]. Therefore, health-care providers must be adequately trained to implement national recommendations and provide specialized breastfeeding support for these mother-infant dyads [11].

Nurses and midwives play a central role across all health systems, constituting approximately half of the professional health workforce [12]. It is estimated that there are around 29 million nurses worldwide and 2.2 million midwives worldwide [13]. The relationship between nursing and midwifery varies considerably across countries; in some countries, midwifery is practised predominantly by nurses, whereas in others nursing and midwifery are regulated as distinct professional groups, including midwives, nurse-midwives or obstetric nurses providing care to women and newborns [12]. The WHO recognises and values the professional distinction between the terms “midwife” and “nurse”, identifying them as distinct occupational groups according to the International Standard Classification of Occupations [14]. At the same time, WHO acknowledges that many countries opt for joint education and regulation of nurses and midwives to respond effectively to health service delivery needs [14]. For the purposes of this review, the term obstetric nurse is used as a functional descriptor to refer to nurses with specialized training in maternal and obstetric care. However, given the international variability in professional education, regulation and scope of practice, this review also includes evidence related to midwives and nurse-midwives when these professionals perform clinical roles equivalent to those of obstetric nurses in the provision of maternal and newborn care. This inclusive approach acknowledges that, while not all midwives are nurses, midwives, nurse-midwives and obstetric nurses frequently share overlapping responsibilities in antenatal care, infant-feeding counselling, shared decision-making and breastfeeding support. Accordingly, the review focuses on functional clinical roles rather than on specific legal or professional titles, in order to comprehensively capture evidence relevant to evidence-based breastfeeding practices in the context of HIV.

In alignment with the Baby-Friendly Hospital Initiative, nurses and midwives play a vital role in protecting, promoting and supporting breastfeeding [15]. As the primary day-to-day service providers, these health professionals should actively participate in delivering evidence-based care to the mothers and infants [15]. In this regard, nurses play a central role in caring for people living with HIV, being in an ideal position to provide effective counselling, advice and support [16]. Through science-based disciplinary knowledge, technical capability and therapeutic relationships, nurses offer a unique contribution to the lived experiences of health and illness [17]. Consequently, the obstetric nurse emerges as a practitioner uniquely positioned to bridge clinical protocols regarding maternal and infant health with patient-centred care. While WHO guidelines provide a robust framework for infant feeding in the context of HIV, translating these recommendations into clinical practice remains complex, as healthcare professionals must balance evolving scientific evidence, residual transmission risk, maternal preferences, and institutional policies. Understanding how obstetric nurses navigate these tensions is essential for strengthening evidence-based practice and improving maternal-infant outcomes. A preliminary search was conducted, and no scoping or systematic reviews of this topic were identified. This scoping review aims to map the existing scientific evidence on the obstetric nurses’ approaches to evidence-based practice regarding breastfeeding in the context of HIV.

## 2. Materials and Methods

This scoping review was conducted in accordance with the Joanna Briggs Institute (JBI) methodology and is reported following the Preferred Reporting Items for Scoping Reviews (PRISMA-ScR) guidelines (PRISMA-ScR checklist in Table S1) [18,19]. Prior to the commencement of the study, the review protocol was registered with the Open Science Framework on 15 September 2025 (https://osf.io/ctf92/overview, accessed on 26 April 2026).

### 2.1. Review Question

The research question was formulated using the PCC (Population, Concept, and Context) framework. The Population (P) comprises obstetric nurses, nurse-midwives, and midwives. The Concept (C) focuses on obstetric nursing practices and interventions related to the implementation of evidence-based breastfeeding guidance within the context of HIV. The Context (C) is defined as maternal and obstetric healthcare services, including antenatal, intrapartum, and postpartum settings.

To enhance clarity and reflect the multifaceted nature of the topic, the review was guided by the following sub-questions:

1. What obstetric nursing practices and interventions are reported in the literature regarding evidence-based breastfeeding for WLWH?
2. What barriers and facilitators influence the implementation of these practices?
3. What implications for clinical practice, education, and policy are identified?

### 2.2. Inclusion Criteria

#### 2.2.1. Participants

The review considered studies focusing on obstetric nurses; however, the inclusion criteria were extended to encompass ‘midwives’ and ‘nurse-midwives’. This decision was justified by the functional overlap and shared clinical competencies of these professionals in the field of maternal and child health, especially as many countries opt for joint regulation of these professions.

#### 2.2.2. Concept

Obstetric nursing interventions in the implementation of evidence-based breastfeeding recommendations for WLWH.

#### 2.2.3. Context

The context included all maternal and obstetric healthcare settings providing care to pregnant, intrapartum, and postpartum WLWH, across hospital-based and community-level services.

#### 2.2.4. Types of Sources of Evidence

This review considered multiple types of evidence, including primary research studies (quantitative, qualitative, and mixed-methods) as well as secondary evidence such as systematic and integrative reviews published in English and Portuguese. Sources of evidence were considered eligible when they addressed the PCC criteria defined for this review, ensuring a comprehensive mapping of the available evidence in accordance with the JBI methodology. The decision to include systematic and integrative reviews was deliberate: given the limited number of primary studies specifically examining obstetric nursing practice in this context, secondary sources allowed for broader mapping of the available evidence landscape. To minimise the risk of evidence amplification, findings from secondary sources that overlapped with primary studies already included in this review were attributed to the original primary source rather than to the review citing it.

### 2.3. Search Strategy

In accordance with the JBI methodology for scoping reviews, a three-step search strategy was undertaken. First, an initial limited search of MEDLINE (via EBSCOhost) was conducted to identify relevant keywords and index terms (e.g., Medical Subject Headings–MeSH) related to the review topic. The text words contained in the titles and abstracts of relevant articles, as well as the index terms used to describe these articles, were analyzed. Second, a comprehensive search strategy was developed. The identified keywords and controlled vocabulary terms were combined using Boolean operators (“AND” and “OR”) and applied across all selected databases. Third, the reference lists of included articles were screened to identify additional relevant studies.

The databases searched included PubMed and Scopus, as well as databases accessed via the EBSCOhost platform, specifically MEDLINE Complete, CINAHL Complete, Cochrane Central Register of Controlled Trials, and Nursing & Allied Health Collection: Comprehensive. The search was limited to studies published in English and Portuguese between 2015 and September 2025. This timeframe (2015–2025) was defined to capture evidence produced around and following the publication of the 2016 WHO guidelines on HIV and infant feeding, which introduced significant updates in clinical recommendations and influenced clinical practice in this field [7].

The full search strategy for all databases was: (“HIV Infections” OR HIV OR “human immunodeficiency virus” OR AIDS) AND (“Breast Feeding” OR breastfeeding OR “breast feeding” OR lactation) AND (Midwifery OR “Nurse Midwives” OR “Obstetric Nursing” OR midwi* OR “nurse midwife*” OR “obstetric nurse*” OR “maternity nurse”) AND (education OR training OR “evidence-based practice” OR “guideline adherence” OR implement* OR “knowledge translation” OR “continuing professional development” OR audit* OR mentorship). The search strategy prioritized terms related to midwives, nurse-midwives and obstetric nurses, reflecting the specific population of interest in this review.

### 2.4. Source of Evidence Selection

All records retrieved from the database searches were imported into Rayyan^®^ (QCRI) for reference management and duplicate removal. Following deduplication, the selection process was conducted in two sequential stages. In the first stage, titles and abstracts were screened for relevance to the review question. In the second stage, the full texts of potentially eligible articles were assessed against the predefined inclusion and exclusion criteria. The screening and eligibility assessment were performed independently by two reviewers. Any discrepancies between reviewers at either stage were resolved through discussion and consensus. When consensus could not be reached, a third reviewer was consulted to support the final decision.

In addition, reference lists of all included studies were manually screened to identify further relevant publications that met the eligibility criteria. Reasons for exclusion at the full-text stage were recorded.

### 2.5. Data Charting and Synthesis of Results

Data were extracted from the included studies using a structured data-charting form developed by the reviewers. The extracted information included author(s), year of publication, country, type of evidence, study design, type of healthcare professionals involved, setting, focus of the study, main results, analytical category, implications for practice and main findings relevant to the review questions.

The data-charting form was pilot-tested on a subset of included studies and refined to ensure consistency and completeness of data extraction. Data extraction was conducted by one reviewer and verified by a second reviewer to ensure accuracy.

Given the heterogeneity of study designs and evidence types, the results were synthesized using a descriptive and thematic approach. Findings were grouped into analytical categories reflecting the roles of obstetric nurses in evidence-based infant feeding practices in the context of HIV, including counselling and decision-making, professional training and implementation of evidence-based practice, and barriers and facilitators influencing clinical practice.

The synthesis aimed to map the range and nature of the available evidence rather than to assess the effectiveness of specific interventions.

## 3. Results

### 3.1. Search Results

**Figure 1:**
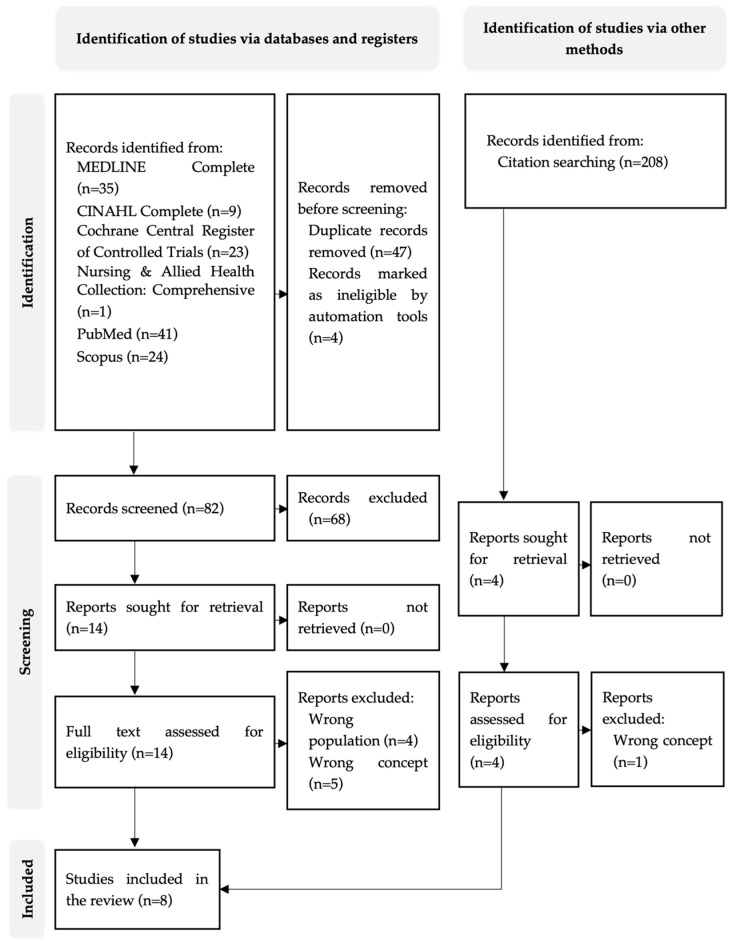
PRISMA-ScR flow diagram of the study selection process.

The study selection process is illustrated in the PRISMA-ScR flow diagram (Figure 1). The search was conducted on 26 August 2025. A total of 133 records were identified through database searching: MEDLINE Complete (*n* = 35), CINAHL Complete (*n* = 9), Cochrane Central Register of Controlled Trials (*n* = 23), Nursing & Allied Health Collection: Comprehensive (*n* = 1), PubMed (*n* = 41), and Scopus (*n* = 24).

Before screening, 47 duplicate records were removed. An additional four records were excluded using automation tools within Rayyan® (QCRI, Doha, Qatar; https://www.rayyan.ai, accessed on 26 August 2025). Following this process, 82 records remained for title and abstract screening. Of these, 68 were excluded for not meeting the inclusion criteria. Fourteen reports were retrieved for full-text assessment. After full-text review, nine reports were excluded due to an incongruent population (*n* = 4) or concept (*n* = 5). Five studies met the eligibility criteria from database searching.

Citation searching of the reference lists of these studies identified 208 additional records. Four full-text articles were assessed for eligibility, of which one was excluded due to an incongruent concept, and three met the inclusion criteria. In total, eight studies were included in this scoping review.

### 3.2. Characteristics of Included Studies

The eight included studies were published between 2015 and 2024. A geographical predominance of sub-Saharan African contexts was observed, particularly in South Africa, Ghana, and Nigeria. One study was conducted in Canada and one survey examined clinical practice across 25 European countries. Two reviews synthesized evidence primarily from sub-Saharan African settings.

The study designs of the eight included studies were heterogeneous and comprised qualitative research, cross-sectional surveys, mixed-methods studies, a case series, a survey examining national guidelines and clinical practice, one integrative review and one systematic review. Across the included studies, four main thematic areas were identified: (1) counselling and decision-making in infant feeding, (2) implementation of evidence-based practice and professional training, (3) barriers and facilitators to evidence-based practice, and (4) implications for obstetric nursing practice. These themes structure the synthesis of findings presented in the following sections.

A detailed overview of the included studies is presented in Table A1.

### 3.3. Counselling and Decision-Making

Across studies, infant-feeding decisions were influenced by a complex interplay of clinical, social, and contextual factors. These factors shape the counselling environment in which obstetric nurses provide guidance on infant feeding for WLWH. Women often navigate concerns about vertical transmission, financial constraints, employment demands, social pressure, and the information provided by healthcare professionals when deciding how to feed their infants [20].

In a South African primary healthcare setting, women living without HIV were significantly more likely to plan breastfeeding than WLWH, reflecting the influence of HIV-related uncertainty on decision-making [20]. Similarly, research conducted in urban Ghana highlighted the critical role of spouses, family dynamics and social acceptance in shaping breastfeeding practices among WLWH [21].

From the provider perspective, counselling practices varied substantially. In Northern Nigeria, only 57.3% of healthcare professionals reported having counselled HIV-positive mothers on infant-feeding options, despite most being aware of available feeding strategies [22]. Feeding options discussed included exclusive breastfeeding, human milk substitutes, heat-treated expressed breast milk, wet nursing and mixed feeding [22]. In South Africa, 45.3% of healthcare workers considered heat-treated expressed breast milk a viable option, with nursing staff demonstrating greater acceptance of this practice [23].

In contrast, the European INSURE survey indicated that, in resource-rich settings, WLWH who wish to breastfeed may be supported when clinically appropriate, often within structured multidisciplinary frameworks [24]. Thirteen participating European countries reported dedicated multidisciplinary approaches involving midwives and clinical nurse specialists [24]. Together, these studies showed that infant-feeding counselling in the context of HIV was influenced by clinical guidance, social pressures, contextual realities, and the confidence of healthcare professionals involved in counselling and support.

### 3.4. Evidence-Based Practice Implementation and Professional Training

Implementation of evidence-based infant-feeding recommendations varied significantly across settings. In Europe, 23 of 25 countries reported having national guidelines on HIV and pregnancy. However, recommendations differed: 12 countries advised against breastfeeding, while 11 permitted breastfeeding under specific clinical conditions [24]. No country offered breastfeeding as an unrestricted option [24]. In Canada, a case series described structured multidisciplinary support for WLWH who chose to breastfeed, involving HIV specialists, obstetricians, midwives and pediatric infectious disease teams [25].

Conversely, studies in sub-Saharan Africa revealed gaps in knowledge translation. In Northern Nigeria, although 75% of healthcare professionals acknowledged exclusive breastfeeding for six months, fewer than half recommended continued breastfeeding thereafter [22]. In South Africa, while 95.3% of respondents were aware of the recommendation for six months of exclusive breastfeeding, only 26.6% knew that breastfeeding could continue up to 12 months, reflecting the emerging evidence at the time of the study [23].

Inconsistent messaging was further reported in a South African mixed-methods study, where frequent changes in global and national guidelines contributed to uncertainty among providers [20]. A systematic review of health-system interventions concluded that specialized training of midwives represents a strategy to strengthen prevention of mother-to-child transmission (PMTCT) service delivery and improve retention [26].

Across the included studies, variability in infant-feeding recommendations and counselling practices was observed across settings, together with inconsistencies in knowledge translation, implementation, and professional training.

### 3.5. Barriers and Facilitators to Evidence-Based Practice

Evidence-based counselling was influenced by both structural and individual-level factors. Barriers included limited specialized knowledge, with only 14.1% of healthcare workers in one South African study considering themselves experts in HIV and infant feeding [23]. Frequent updates to international and national guidelines contributed to inconsistent counselling practices [20]. An integrative review identified that personal beliefs among providers sometimes overrode official recommendations, resulting in contradictory counselling messages [27]. Additional challenges included skepticism regarding the feasibility of exclusive breastfeeding, concerns about milk sufficiency, maternal employment constraints, burnout and stress [27]. Social stigma also emerged as a significant barrier. In Ghana, some women reported experiencing hostility from midwives in maternity units due to their HIV-positive status [21].

Facilitators included awareness of HIV transmission risks (86.6% in one Nigerian study) and generally positive professional attitudes toward breastfeeding when aligned with updated guidelines [22,23]. Encounters with trained HIV counsellors significantly encouraged mothers to continue breastfeeding until the appropriate time for cessation [21].

Taken together, these barriers and facilitators demonstrate that effective evidence-based counselling depends not only on knowledge acquisition but also on organizational stability, supportive supervision and the professional empowerment of obstetric nurses and midwives.

### 3.6. Implications for Obstetric Nursing Practice

Across the included studies, obstetric nurses and midwives were described as participating in counselling, breastfeeding support, and multidisciplinary care. In resource-rich settings, multidisciplinary collaboration was reported as an organizational strategy [24,25].

Timing of counselling was also identified as relevant. Although 77.9% of providers in a Nigerian tertiary facility believed that infant-feeding counselling should begin during prenatal visits, fewer than half reported initiating counselling at that stage [22]. Collaborative and supportive counselling approaches were also reported in the literature [27].

Confidence in practical breastfeeding skills was generally high among South African healthcare workers (73.4%), with nurses demonstrating slightly greater confidence than other professionals, although differences in confidence levels were observed between professional groups, these were not statistically significant, suggesting no meaningful differences between groups [23].

## 4. Discussion

As a scoping review, this study aimed to map the available evidence on obstetric nursing interventions supporting the interpretation and implementation of evidence-based breastfeeding recommendations for WLWH. Following established scoping review methodology [18,19], the discussion presents an analytical synthesis grounded in patterns, gaps, and tensions identified across the included studies, complemented by interpretative reflection informed by the broader literature on knowledge translation and professional practice. The limited number of eligible studies reflects the scarcity of research specifically examining obstetric nursing practice in HIV-related infant feeding, as most available studies aggregate nurses with other healthcare professionals. This review contributes to the literature by not only mapping variability in infant-feeding practices but also by identifying a critical and underexplored gap: the lack of profession-specific evidence on obstetric nursing practice in this field. This gap limits the development of targeted training, policy, and research agendas.

The interpretative framework adopted in this review is used as an analytical lens to support the interpretation of patterns identified across the included studies. These concepts are not presented as direct empirical findings but as theoretical interpretations that may help explain observed inconsistencies in counselling, guideline uptake, and professional practice. This distinction is particularly important given the limited number and heterogeneity of the included studies. Such an approach is consistent with the purpose of scoping reviews, which aim not only to map existing evidence but also to identify structural gaps and generate conceptually informed insights relevant to clinical practice, professional development, and future research.

### 4.1. The Central Challenge

The findings of this review, particularly the inconsistencies in counselling practices, gaps in guideline uptake, and the influence of personal beliefs identified across the included studies, suggest that the implementation of HIV-related breastfeeding guidance may not be fully explained by a simple knowledge deficit. Rather, these patterns may reflect challenges in how obstetric nursing practice translates evolving evidence into context-sensitive and ethically coherent care.

Across the eight included studies, the coexistence of knowledge gaps, inconsistent counselling practices, and variability in guideline application indicates that the availability of recommendations alone does not ensure their consistent implementation. These patterns may be interpreted through three interrelated dimensions.

First, obstetric nurses operate within clinical contexts that require ongoing negotiation of multiple priorities, including the prevention of MTCT, the recognized benefits of breastfeeding, and the woman’s right to informed decision-making. This negotiation is shaped by contextual factors such as resource availability, institutional protocols, and sociocultural influences.

Second, the interaction between institutional constraints, evolving clinical guidance, and professional responsibility may give rise to situations that resemble dynamics described in the literature as moral distress, particularly when there is a perceived misalignment between knowledge, clinical judgement, and the ability to act accordingly [28,29]. Although this concept was not explicitly reported in the included studies, it may offer a useful interpretative perspective for understanding the tensions identified.

Third, the rapid evolution of international recommendations, from discouraging breastfeeding to supporting exclusive breastfeeding with antiretroviral therapy, and more recently to shared decision-making approaches, may contribute to forms of epistemological uncertainty among healthcare professionals. This may be reflected in fragmented knowledge and inconsistencies in counselling practices across different settings [20,22,23,27].

Taken together, these findings suggest that the implementation of evidence-based breastfeeding guidance in the context of HIV may be influenced not only by access to knowledge but also by the organizational and professional conditions identified across the included studies, which shape how evidence is interpreted and applied in practice.

### 4.2. The Global Dichotomy: Resource-Limited vs. Resource-Rich Settings

#### 4.2.1. Findings from the Included Studies

The evidence mapped in this review suggests a global divergence in infant-feeding practices within the context of HIV, shaped by differences in socioeconomic conditions and healthcare infrastructures. In Southern and Eastern Africa, where HIV remains a major contributor to child mortality and where infectious diseases and malnutrition are prevalent, exclusive breastfeeding continues to be described as a critical survival strategy [7].

Within these settings, the included studies indicate challenges in implementing evidence-based recommendations, including knowledge gaps regarding updated protocols [20,23], discrepancies between theoretical knowledge and clinical practice [20], and counselling influenced by personal beliefs or contextual pressures rather than by scientific evidence [20,27]. These findings suggest that, in high-prevalence and resource-limited settings, the implementation of infant-feeding recommendations is influenced not only by the dissemination of guidelines but also by the professional and institutional conditions that shape how they are interpreted and applied in practice.

#### 4.2.2. Recent Literature Beyond the Included Studies

In contrast, evidence from resource-rich settings suggests a gradual shift in practice. Earlier recommendations in high-income countries generally discouraged breastfeeding due to the risk of MTCT. However, more recent literature indicates increasing recognition of shared decision-making in supporting WLWH who wish to breastfeed under appropriate clinical conditions [24,25]. Emerging evidence suggests that when viral suppression is maintained, and when close clinical monitoring and specialized lactation support are available, breastfeeding may be considered within carefully supervised care frameworks [30].

#### 4.2.3. Critical Interpretation: Risk Negotiation in Context

This divergence illustrates how infant-feeding decisions in the context of HIV are deeply embedded in local epidemiological and health-system realities. In resource-limited contexts, the health risks associated with avoiding breastfeeding, including increased infant mortality due to malnutrition and infection, may exceed the residual risk of MTCT under effective ART. Conversely, in resource-rich settings, the availability of safe alternatives and intensive monitoring systems allows greater emphasis on maternal autonomy and informed choice.

Nevertheless, the studies included in this review suggest that nurses across both contexts often report insufficient training, inconsistent institutional guidance, and limited decision-making autonomy when translating guidelines into individualized care [20,23,26]. These findings indicate that the central challenge may lie less in the availability of resources and more in the professional capacity to interpret and apply evolving evidence within complex clinical environments.

### 4.3. The Role of Obstetric Nurses: Professional Identity and Knowledge Translation

For this review, the term obstetric nurse was used as a functional descriptor encompassing nurses, nurse-midwives, and midwives who perform comparable clinical roles in maternal and newborn care. This inclusive definition reflects the considerable international variation in professional regulation and educational pathways while recognising the shared responsibilities of these professionals in antenatal care, infant-feeding counselling, and breastfeeding support. Despite this recognised professional mandate, the findings of this review suggest that challenges in knowledge translation often characterise obstetric nursing practice in the context of HIV-related infant feeding. The included studies suggest that, in the available evidence, knowledge translation challenges affecting obstetric nurses and midwives are often reported within broader multidisciplinary groups rather than as profession-specific findings. The lack of global uniformity in professional titles, scopes of practice, and regulatory frameworks may further complicate the standardised implementation of evidence-based guidelines. Variability in professional roles and decision-making authority across healthcare systems may contribute to inconsistencies in how recommendations are interpreted and applied in practice.

These observations align with broader research on evidence-based practice, which emphasises that the uptake of scientific evidence is shaped not only by individual competence but also by organisational culture, professional hierarchies, and the degree to which nurses are empowered to participate in clinical decision-making processes [31,32]. Consequently, strengthening evidence-based breastfeeding support in the context of HIV requires attention not only to individual knowledge acquisition but also to the professional and institutional environments in which nursing practice is situated.

### 4.4. Counselling as an Ethical and Clinical Practice

The variability in counselling practices observed across the included studies suggests that infant-feeding counselling in the context of HIV is not consistently grounded in evidence-based guidance. Rather, it may be influenced by gaps in knowledge regarding updated WHO recommendations, as well as by personal beliefs and uncertainties among healthcare providers [20,23,27]. These factors contribute to inconsistencies in the information provided to women and may affect decision-making processes.

The findings also indicate that counselling is not solely a technical activity but involves complex interpersonal and ethical dimensions. The importance of initiating counselling during pregnancy and adopting collaborative, non-judgmental communication approaches reflect the need to support informed and autonomous decision-making [22,27].

Recent literature from high-resource settings further reinforces the value of multidisciplinary counselling frameworks, in which obstetric nurses and midwives play a central role in facilitating continuity of care and supporting women throughout pregnancy and the postpartum period [24,25,33]. Within these models, counselling is understood not only as the transmission of information but also as an ethical practice that balances maternal autonomy with clinical risk.

### 4.5. Clinical Lactation Support and Prevention of Complications

The evidence reviewed suggests that nurses play a key role in providing practical breastfeeding support, particularly in positioning, attachment, and milk expression [23]. These competencies are clinically important for preventing complications such as mastitis and nipple trauma, which may increase the risk of viral transmission. Beyond technical skills, effective lactation support may also strengthen maternal confidence and self-efficacy, which are critical for sustaining breastfeeding practices among WLWH.

Evidence from high-resource healthcare systems further indicates that specialised midwifery-led lactation support, combined with structured clinical monitoring, may enhance breastfeeding outcomes while maintaining safety in carefully supervised contexts [30,34].

However, a recurring limitation within the literature is the tendency to group nurses together with other healthcare professionals under the broad category of “healthcare providers.” This aggregation obscures the specific contributions of nursing practice and limits the identification of profession-specific competencies, training needs, and areas of expertise. Accordingly, the limited number of eligible studies and the frequent aggregation of nurses within multidisciplinary samples highlight a significant gap in the literature, namely the lack of profession-specific evidence addressing obstetric nursing practice in HIV-related infant feeding.

### 4.6. Bridging the Gap: Training and Professional Development

The findings of this review highlight the importance of ongoing professional development to ensure that clinical practice remains aligned with evolving evidence. The included studies suggest that specialised training programmes play an important role in strengthening PMTCT services and improving counselling practices [26]. However, the evidence also indicates that training should address not only clinical protocols but also the personal beliefs and social stigmas that may influence counselling practices [20,27].

Recent literature further emphasises the need for continuous professional education for midwives, particularly in relation to communication skills and postpartum care within the HIV context [35]. Proposed educational strategies include integrating HIV-related content into undergraduate and postgraduate curricula, promoting interdisciplinary learning environments, and incorporating simulation-based and case-based learning approaches. These strategies support the development of adaptive clinical reasoning and prepare healthcare professionals to manage complex clinical and ethical situations [35].

Importantly, the challenges identified in this review suggest that training should extend beyond technical knowledge to include reflective practice, ethical reasoning, and interprofessional collaboration. Such competencies are essential for navigating the tensions that arise when clinical guidelines, institutional policies, and patient preferences intersect in real-world practice [36,37]. These findings also highlight the importance of institutional frameworks that actively support and protect nursing autonomy within shared decision-making processes, enabling obstetric nurses to translate evidence into practice in a consistent and ethically grounded manner.

In practical terms, this includes: (i) integrating HIV-specific infant-feeding guidance into undergraduate and postgraduate nursing curricula; (ii) implementing structured supervision and mentorship models to support consistent counselling practices; and (iii) establishing clear institutional protocols that align clinical guidelines with decision-making pathways, thereby reducing variability in practice.

### 4.7. Heat-Treated Expressed Human Milk

The evidence in this review indicates that heat-treated expressed human milk is recognised as a potential infant-feeding option in specific clinical circumstances, such as maternal illness, temporary interruption of ART, or neonatal vulnerability [7,23]. However, its use appears to be limited and context dependent.

The current evidence base provides limited data regarding the feasibility and sustainability of implementing this approach in community settings. While international guidance acknowledges its potential role in certain situations, further implementation research is needed to assess its safety, acceptability, and practical applicability outside controlled clinical environments [7,38].

These findings suggest that, although technically recognised, this strategy remains insufficiently explored in practice, highlighting the need for further research addressing contextual feasibility, training requirements, and integration into clinical care pathways.

Overall, the findings of this review indicate that the implementation of evidence-based breastfeeding guidance in the context of HIV is shaped by a combination of clinical, organisational, and contextual factors. While variability in counselling practices and gaps in knowledge translation were identified, the evidence also highlights a significant gap in the literature, namely the limited availability of profession-specific research addressing obstetric nursing practice. This gap constrains the development of targeted training, policy, and research strategies.

### 4.8. Limitations

This review presents several limitations that should be considered when interpreting the findings. First, the number of included studies was limited (*n* = 8), and the evidence base was characterised by methodological heterogeneity, including qualitative studies, surveys, and reviews. This limits the comparability of findings and the strength of conclusions that can be drawn.

Second, a geographical concentration of studies in sub-Saharan Africa was observed, which may limit the transferability of findings to other healthcare contexts, particularly resource-rich settings.

Third, a recurring limitation within the included studies was the aggregation of nurses within broader categories of “healthcare providers,” which restricted the ability to identify profession-specific practices, competencies, and challenges in obstetric nursing.

Finally, the rapidly evolving nature of international guidelines on HIV and infant feeding may influence the interpretation of findings, particularly in studies conducted under earlier recommendations.

Additionally, this review included both primary studies and secondary evidence (one integrative and one systematic review). While this approach was intended to support comprehensive mapping in a field with limited profession-specific primary research, it introduces a risk that findings from primary studies may be indirectly amplified through secondary sources. Steps were taken to mitigate this by attributing overlapping findings to their primary sources; nonetheless, this methodological choice and its potential implications for the breadth and weight of the mapped evidence should be considered when interpreting the results.

These limitations highlight the need for further research that is methodologically robust, geographically diverse, and explicitly focused on obstetric nursing practice.

## 5. Conclusions

This scoping review mapped the available evidence on obstetric nursing practice in relation to evidence-based breastfeeding in the context of HIV. The findings suggest that variability in counselling practices, inconsistencies in guideline implementation, and the influence of contextual and professional factors shape how evidence is translated into clinical care.

Obstetric nurses occupy a key position in mediating between clinical guidance and women’s lived experiences. However, their capacity to do so is influenced by the organisational and professional conditions in which they practice, including training pathways, institutional support, and opportunities for participation in clinical decision-making.

Importantly, this review highlights a critical gap in the literature, namely the limited availability of profession-specific evidence addressing obstetric nursing practice. Addressing this gap requires not only strengthening knowledge translation, but also promoting context-sensitive, ethically informed, and discipline-specific approaches to practice.

Future research should prioritise longitudinal, geographically diverse, and profession-focused studies to better understand and support the role of obstetric nurses in infant-feeding decision-making among WLWH.

## Data Availability

No new data were created or analyzed in this study. Data sharing is not applicable to this article.

## Supplementary Materials

The following supporting information can be downloaded at: https://www.mdpi.com/article/10.3390/healthcare14091172/s1, Table S1: PRISMA-ScR Checklist.

## Abbreviations

The following abbreviations are used in this manuscript:

HIV	Human immunodeficiency virus
WLWH	Women living with HIV
WHO	World Health Organization
MTCT	Mother-to-child transmission
ART	Antiretroviral therapy
JBI	Joanna Briggs Institute
PRISMA-ScR	Preferred Reporting Items for Scoping Reviews
PCC	Population, Concept and Context
PMTCT	Prevention of mother-to-child transmission

## Appendix A

**Table A1:** Characteristics of included studies and mapping to analytical categories.

Author, Year	Country	Type of Evidence	Study Design	Population/ Professionals	Setting	Focus of the Study	Main Results	Key Findings for Obstetric Nursing Role	Analytical Category	Implications for Practice
West et al., 2019 [20]	South Africa	Primary	Mixed methods	Healthcare providers including nurses and nurse-clinicians	Primary healthcare setting	Infant feeding counselling and maternal decision-making	Infant feeding decisions were driven by a combination of fear of vertical HIV transmission, employment, financial constraints, social pressure, and health care providers advice.	Inconsistent counselling due to conflicting guidelines; counselling strongly influenced women’s feeding decisions	Counselling and decision-making; Barriers and facilitators	Need for consistent, evidence-based counselling frameworks
Acheampong et al., 2017 [21]	Ghana	Primary	Qualitative	Midwives, counsellors	Public hospital	Social and professional influences on breastfeeding decisions	Breastfeeding decisions were influenced by their social circles and healthcare interactions.	Midwives’ advice shaped women’s feeding practices; counsellors encouraged breastfeeding	Counselling and decision-making; Barriers and facilitators	Training in stigma-free, evidence-based communication
Nashid et al., 2020 [25]	Canada	Primary	Observational descriptive study (Case series)	Multidisciplinary team including midwives	High-resource clinical setting	Shared decision-making in infant feeding	Maintaining adherence to HIV care and undetectable viral loads, two women successfully breastfed their infants under the guidance of multidisciplinary team. All three infants remained HIV-negative.	Midwives supported informed choice through individualized counselling and viral load monitoring	Counselling and decision-making	Support shared decision-making models in clinical practice
Ambia & Mandala, 2016 [26]	Sub-Saharan Africa	Secondary	Systematic Review	Midwives, nurses	PMTCT services	Interventions to improve PMTCT outcomes	Social, behavioural, and structural interventions with the potential to improve PMTCT outcomes.	Training of midwives and integration of services improved counselling and infant outcomes	Evidence-based practice implementation and training	Investment in structured professional training programmes
Janse van Rensburg et al., 2016 [23]	South Africa	Primary	Cross-sectional survey	Healthcare workers including nurses	Regional hospital	Knowledge and practices regarding infant feeding	Healthcare workers show knowledge gaps regarding basic concepts and practices. Although not statistically significant, nurses expressed more confidence in demonstrating breastfeeding techniques than other healthcare professionals.	Nurses showed gaps between guidelines and practice, particularly regarding breastfeeding recommendations	Evidence-based practice implementation and training; Barriers and facilitators	Ongoing education aligned with updated WHO guidelines
Iliyasu et al., 2020 [22]	Nigeria	Primary	Cross-sectional survey	Healthcare professionals including nurses and midwives	Tertiary health facility	Knowledge and counselling practices	Healthcare professionals exhibit knowledge gaps and misconceptions regarding infant feeding practices.	Limited knowledge and delayed counselling on infant feeding options among nurses and midwives	Evidence-based practice implementation and training; Counselling and decision-making; Barriers and facilitators	Strengthen early and structured counselling training
Tuthill et al., 2015 [27]	Multiple countries	Secondary	Integrative review	Healthcare providers including nurses/ midwives	PMTCT	Provider-level challenges in infant feeding counselling	Counseling was based on personal experiences or beliefs rather than official guidelines. Healthcare providers also experienced a lack of practical strategies to support mothers.	Providers’ beliefs and fear of transmission often overrode guidelines	Barriers and facilitators; Counselling and decision-making	Address personal beliefs through reflective and evidence-based education
Keane et al., 2024 [24]	Europe	Primary	Survey of guidelines and practice	Healthcare professionals	European maternity services	Guideline variability and clinical practice	While 23 out of 25 countries have established guidelines on HIV and pregnancy, significant discrepancies in recommendations regarding breastfeeding have been identified.	Heterogeneity in recommendations limited professionals’ confidence in counselling		
